# Whole-genome sequence and pathogenicity of a fowl adenovirus 5 isolated from ducks with egg drop syndrome in China

**DOI:** 10.3389/fvets.2022.961793

**Published:** 2022-08-12

**Authors:** Hao Chen, Meng Li, Siyu Liu, Juan Kong, Dan Li, Jiaxun Feng, Zhixun Xie

**Affiliations:** ^1^College of Life Science and Technology, Guangxi University, Nanning, China; ^2^Guangxi Key Laboratory of Veterinary Biotechnology, Guangxi Veterinary Research Institute, Nanning, China; ^3^College of Life Science, Qufu Normal University, Qufu, China

**Keywords:** fowl adenovirus 5, egg drop syndrome, phylogenetic analysis, pathogenicity, duck

## Abstract

Recently, fowl adenovirus (FAdV) infection has become widespread in poultry in China and may be asymptomatic or associated with clinical and other pathological conditions. In 2017, a severe egg drop syndrome outbreak in breeder ducks (45 weeks old) occurred in eastern Shandong province in China. The egg production rate declined from 93 to 41%, finally increasing to ~80% (did not reach complete recovery). The presence of the virus was confirmed by FAdV-5 specific PCR assay, and it was designated strain WHRS. Furthermore, next-generation and Sanger sequencing of genomic fragments yielded a 45,734 bp genome. Phylogenetic analysis showed that the genomic sequence of the WHRS strain was most homologous-(99.95%) to that of the FAdV-5 17/25,702 and 14/24,408 strain, sharing 32.1~53.4% similarity with other FAdV strains in the genus *Aviadenovirus*. Infected duck embryos died within 3–5 dpi, but no deaths occurred in the infected ducks. Strain WHRS could cause egg drop syndrome in ducks, accompanied by clinical signs similar to those of natural infections. Overall, strain WHRS is lethal to duck embryos and causes egg drop syndrome in breeder ducks.

## Introduction

Fowl adenoviruses (FAdVs) belong to the genus *Aviadenovirus* in the family *Adenoviridae*, which contains five genera (*Mastadenovirus, Aviadenovirus, Atadenovirus, Siadenovirus*, and *Ichtadenovirus*). The genus *Aviadenovirus* is split into five species (FAdV-A to FAdV-E) based on restriction enzyme digestion characteristics (1). FAdVs have been divided into 12 serotypes (FAdV-1 to 8a and FAdV-8b to 11) based on the results of cross-neutralization tests and restriction enzyme digestion patterns (2, 3). The 12 serotypes have been grouped into five FAdV species currently recognized as follows: FAdV-A (FAdV-1), FAdV-B (FAdV-5), FAdV-C (FAdV-4 and FAdV-10), FAdV-D (FAdV-2, FAdV-3, FAdV-9 and FAdV-11) and FAdV-E (FAdV-6, FAdV-7, FAdV-8a and FAdV-8b) (4). FAdVs are nonenveloped icosahedral viruses with a diameter of ~70~100 nm. The major structural proteins are hexon, fiber, and the penton base (5, 6). Due to the complexity of cross-neutralization testing, phylogenetic analyses based on the loop 1 (L1) region of the hexon gene have been applied for the identification and differentiation of at least 12 serotypes within the five species (3, 7).

FAdVs cause different symptoms, including inclusion body hepatitis (IBH), hepatitis-hydropericardium syndrome (HHS), and gizzard erosions in poultry, and some nonpathogenic FAdVs cause no obvious lesions (8–11). Although the results of postmortem examinations of birds with the three symptoms have varied, several pathological findings were similar, including an enlarged and friable liver with necrotic foci, lymphocytolysis and cyst formation in the spleen and bursa of Fabricius, and atrophy in the thymus. Moreover, there are other clinical symptoms in the FAdV-infected birds, such as severe acute respiratory symptoms, lameness, and swelling of the tarsal joints (12–15). In addition, members of the genus *Aviadenovirus* have been isolated from ducks, geese, turkeys, falcons, pigeons, and psittacines (16–20).

FAdV-5 infection causes lameness, swelling of the tarsal joints, and IBH in broilers, and the FAdV-5 strain was isolated from a healthy mallard duck (21). The chicken antiserum against FAdV-5 reference strain 340 could not neutralize any of the newly isolated viruses (22). In 2017, an outbreak of severe egg drop syndrome with IBH in breeder ducks (45 weeks old) occurred in eastern Shandong Province in China. The egg production rate declined to 40% of the normal production level and then increased to ~80%. Viral nucleotides were detected using (RT)-PCR, and tests for tembusu virus, EDSV (egg drop syndrome virus), and avian influenza A virus, which could cause a drop in egg production, were negative. In the present study, we confirmed the infectious agent of the disease using virus isolation, next-generation sequencing, phylogenetic analysis and pathogenicity testing.

## Materials and methods

### Ethics statement

The present study was approved by the Animal Ethics Committee of the Guangxi Veterinary Research Institute (license number: 20200509). Animals were kept and samples were conducted based on the protocol #2019C0406 issued by Animal Ethics Committee of Guangxi Veterinary Research Institution.

### Clinical samples

The laying rate decreased from >90 to ~40% within 20 days, and hyponoia and anorexia were observed in most breeder ducks. Egg production gradually increased and recovered in the subsequent month. A total of 16/6,000 ducks died during the outbreak. The dead ducks and 20 female ducks with clinical symptoms that had stopped laying eggs were used for aetiological analysis. Nutritional (calcium and phosphorus) and aflatoxin factors were excluded by investigation and testing. Blood agar plates were prepared for sterile isolation of bacteria from the liver, follicles and brain and cultured at 37°C under conventional or facultative anaerobic conditions. The postmortem examinations of the diseased ducks included liver enlargement, congestion, theca folliculi rupture, and even yolk peritonitis in individual diseased ducks. Liver and theca folliculi samples were collected from a breeder duck farm in Shandong province, frozen and transported to our laboratory on dry ice.

### Virus propagation

Viral nucleic acid was extracted from samples to detect duck viruses, as assessed by (RT-)PCR assays (23–27). Matrix genes (a pair of specific primers, F:5-TTCTAACCGAGGTCGAAAC-3, R: 5-AAGCGTCTACGCTGCAGTCC-3) were applied for AIV detection. Tissue homogenates (10%) were freeze–thawed three times and then centrifuged at 8,000 × g for 15 min. Primary cells were collected from 11-day-old duck embryos. The supernatant was filtered through a 0.22-μm pore filter and then added to 80% monolayer duck embryo hepatic (DEH) cell and duck embryo fibroblast (DEF) cell cultures, which were grown in Dulbecco's modified Eagle's medium/Ham's F12 medium (DMEM/F12) (HyClone, Waltham, MA, USA) supplemented with 1% fetal bovine serum (FBS) (HyClone). Tests for AIV, TMUV, FAdV, EDSV, DHAV-I, DPV and DRV were negative. The virus was blindly passaged for serial generations on DEH monolayer cells. The cytopathic effects (CPEs) were monitored daily, and the cell culture was harvested when the CPE reached 80%. The viral titres were determined to be 10^4.0^ median embryo lethal dose (ELD_50_)/0.2 ml using the Reed and Muench method (28).

### Nucleic acid extraction

Based on the appearance of the CPEs, we detected duck reovirus and fowl adenovirus (29, 30). To obtain high-quality sequencing data, cell culture supernatants were centrifuged at low speed (6,000 × g, 10 min) to remove the precipitate, and then the supernatant was centrifuged at high speed (24,000 × g, 3 h). The pelleted cell-free virions were used for RNA/DNA isolation. RNA was reverse transcribed into cDNA. The DNA concentration was determined with a NanoDrop spectrophotometer.

### Whole genome sequencing of the WHRS strain

Whole-genome sequencing was carried out using the HiSeq2500 platform. DNA was disrupted into homogenous fragments by ultrasonication. A paired-end library with a 200-bp insert size was generated according to the manufacturer's protocol (TIANSeq DirectFast DNA Library Prep Kit), and barcodes were used to sequence the virus samples in a single lane. The reads corresponding to different strains were separated based on a perfect match to the barcode sequence. As the virus was passaged in DEH, the genome sequence of *Anas platyrhynchos* should be removed to obtain clean data. Therefore, we initially mapped all reads against the available genome of BGI duck 1.0 and twinkle mtDNA (XM_021279514) and used only unmapped reads for the assembly of the virus genomes.

The next-generation sequencing raw reads were analyzed and assembled into contiguous sequences (contigs) using CLC Genomics Workbench 10.0 software (CLC Bio). All virus-aligned contigs (≥500 bp) were identified and extracted after alignment to the reference genome (ftp://ftp.ncbi.nlm.nih.gov/blast/db/ref_viruses_rep_genomes.tar.gz). The scaffolding and ordering of the contigs for each segment were facilitated by mapping the contigs on the reference virus genome FAdV 340 (KC493646) (21).

Contigs were manually aligned according to the genomic sequence of the FAdV strain 340. Primers (cF: 5′- TCATAGGGTGAACCTTCTT-3′, cR: 5′- TGTCGATGTAGGTTGAGTCA-3′) were designed by Oligo 7.0 based on the adjacent contig terminals and utilized to amplify the intermediate sequences. Multiple segments were assembled into two long fragments. The amplified fragments were determined using Sanger dideoxy sequencing (Sangon, Shanghai, China). Finally, the whole genome of the WHRS strain was manually assembled using the Seqman program implemented in Lasergene (version 7.0; DNASTAR Inc., Madison, WI, USA) and deposited into the GenBank database (accession number: OM836676).

### Sequence analysis

Prediction of the open reading frames (ORFs) and genes was carried out using GeneMark software (http://topaz.gatech.edu/GeneMark/heuristic_gmhmmp.cgi), and sequence alignments and analyses were performed using MEGA 5.0 (30). ORFs coding for peptides with ≥30 amino acids were identified as potential protein-coding ORFs. Splice acceptor and donor sites were manually predicted based on previous findings in other FAdV genomes. ORFs and putative proteins were compared to proteins in the NCBI GenBank database using BLASTP.

### Phylogenetic analysis

The complete genomic sequence of the FAdV isolate WHRS was aligned with other FAdV genomic sequences available in the GenBank database to determine the nucleotide sequence homologies using ClustalW in MEGA 5.0 (31). Multiple-sequence alignment of deduced amino acid sequences of the hexon and penton proteins was performed using ClustalW. A phylogenetic analysis of complete genome sequences was performed using the maximum-likelihood (ML) method in MEGA 5.0. The phylogenetic trees of the hexon and penton proteins were constructed using the neighbor-joining (NJ) method of MEGA 5.0, with absolute distances based on 1,000 bootstrap replicates.

### Detection of FAdV-5 by PCR assay

A pair of FAdV-5-specific primers (dF: 5′-ATTAGCAACATCGCAGAGT-3′, dR:5′-AAGTCGTAGTGGAAGTAGTG-3′) was designed using Primer Premier v6.0 to yield a PCR product of 977 bp. The total DNA of the diseased duck liver and the viral stock were extracted using the phenol–chloroform method and used as a template. The amplicons were visualized on a 1% agarose gel.

### Experimentally induced infection with the WHRS strain in breeder ducks

To evaluate the pathogenicity in ducks, thirty 400-day-old breeder ducks were subcutaneously inoculated with 10^4.0^ ELD_50_ of the WHRS strain, whereas 30 ducks were infected with sterile PBS and served as the negative controls. All animals were free of FAdVs and other egg drop-related viruses, as determined by RT–PCR assays of oral and cloacal swabs. All animals were kept separately in specific pathogen-free chicken isolators. Clinical signs and egg production were monitored daily for 30 days. The cloacal swabs were collected from infected ducks to detect viral shedding by PCR assay using dF/dR.

## Results

### Clinical features, virus isolation and detection

**Figure 1:**
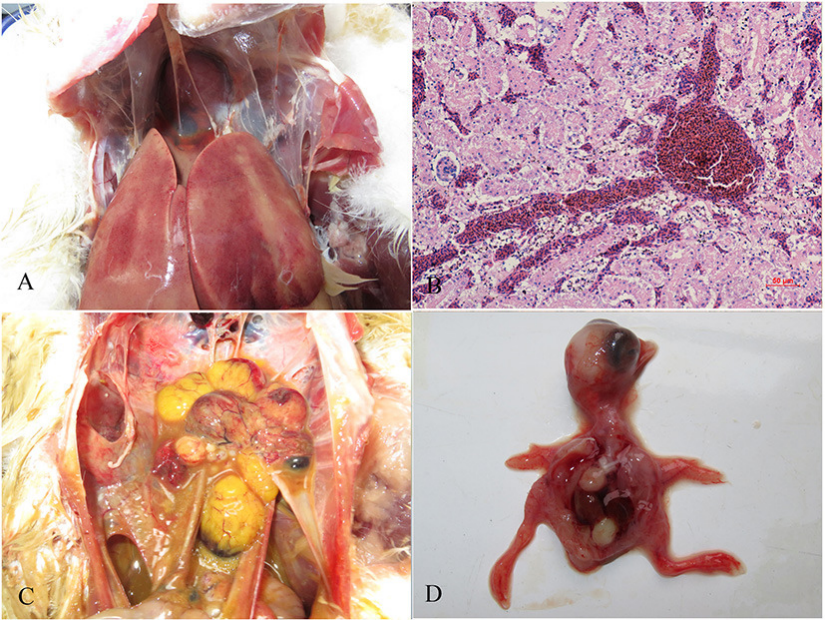
Gross lesions and histopathologic changes in ducks and embryos. **(A)** Lesions in the liver, swelling, and congestion; **(B)** necrosis and fatty degeneration of hepatocytes, and intranuclear inclusions in liver parenchymal cells; **(C)** lesions in the ovary, follicular dysplasia, necrosis, and rupture; and **(D)** an infected embryo with subcutaneous hemorrhage.

**Figure 2:**
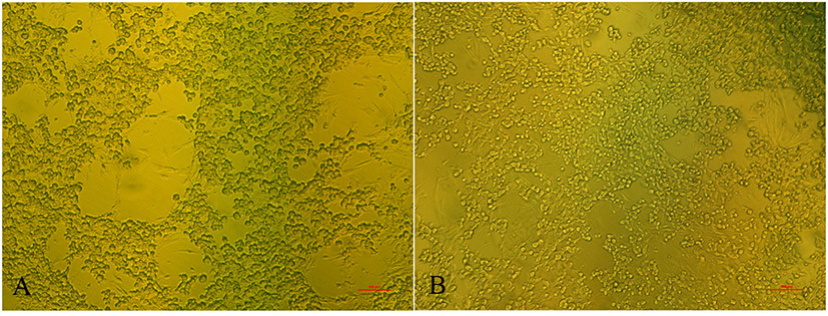
The cytopathic effects in DEH cells. The elongated cells are fibroblasts. **(A)** DEH cell aggregation and fusion after infection with WHRS; **(B)** normal DEH cells in the control group. The scale bar is 100 μm.

There were no obvious clinical symptoms, except that egg production declined and then recovered during a 2-month period in the breeder duck flock. Twenty layer ducks, which had stopped laying eggs, were used for postmortem examination and etiology determination. The postmortem examinations revealed severe gross lesions, such as an enlarged and fragile liver with a pale color and steatosis, as well as ruptured, haemorrhagic, and necrotic follicles (Figures 1A,C). Mild gross lesions, such as oedema, lung congestion, nephritis, and enteritis, also appeared in diseased ducks. The livers and theca folliculi were homogenized for virus isolation and DNA extraction. (RT-)PCR assay for common duck viruses were negative. The main histopathological changes in the liver of diseased ducks included severe hemorrhage, necrosis and fatty degeneration of hepatocytes, intranuclear inclusions in liver parenchymal cells, and lymphocyte infiltration in the liver (Figure 1B). The embryos died at 72~108 h post-infection after three serial passages. Severe subcutaneous hemorrhage was observed in the dead embryos and was complicated by hepatitis (Figure 1D). The viral titer of the viral stock was 10^4.7^ ELD_50_/ml. A severe CPE was observed in DEH cells in cases of primary infection, but no CPE was observed in DEF cultures inoculated with the WHRS strain (Figure 2). The field liver sample and viral stock were positive for FAdV-5 according to the PCR assay.

### Genomic sequencing and phylogenetic analyses

After filtering for contaminating duck chromosomal sequences, the average coverage for all sequenced genomes was between 200 and 40,000 reads per nucleotide. *De novo* assembly using 7% of the reads was optimal, resulting in two major contigs of 39,154 bp and 4,244 bp. Based on the reference genomic sequences, PCR amplification and Sanger sequencing of the interval and terminal fragments yielded a final genome of 45,734 bp. The G + C content of the WHRS strain genome was 56.55%. The inverted terminal repeat (ITR) was considered 81 bp long (with a 5 bp deletion compared with a 340 bp deletion). The WHRS strain shared genomic sequence identities of 32.1~98.6% with other *Aviadenovirus* strains, andit shared a high sequence identity (99.95%) with the FAdV-5 17/25702 and 14/24408 strain. WHRS shared low sequence identities (32.1% and 52.6%) with FAdV-4 and FAdV-8b, respectively, which are prevalent in China and have caused IBH and HPS in poultry flocks since 2014. Compared with the deduced proteins of strain 340, the most amino acid deletions/insertions were observed in 100 K (9 amino acid insertion, 195D, 234S, 316-318GGG, 332-335NAAR). Moreover, amino acid deletion/insertion occurred in dUTPase, penton, pVI, ORF19A, DNA polymerase, DNA-binding protein, ORF22A, ORF20A and OFR19.

**Figure 3:**
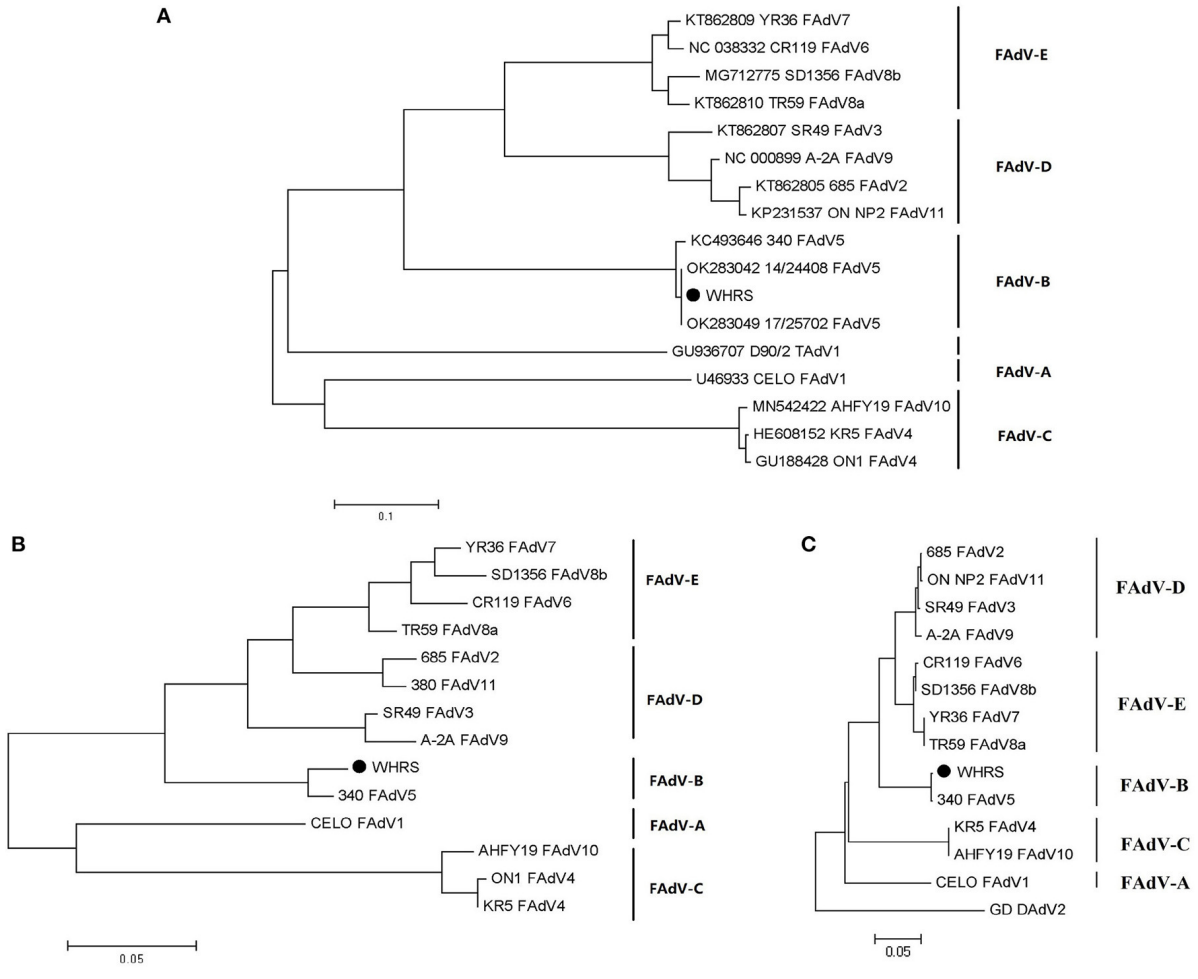
**(A)** Phylogenetic tree constructed using the ML method based on the genomic sequences of the WHRS strain (•) and reference FAdV strains. **(B)** Phylogenetic tree constructed using the NJ method based on the amino acid sequences of hexon proteins of the WHRS strain (•) and other FAdV strains. **(C)** Phylogenetic tree constructed using the NJ method based on the amino acid sequences of the penton proteins of the WHRS strain (•) and other FAdV strains.

**Figure 4:**
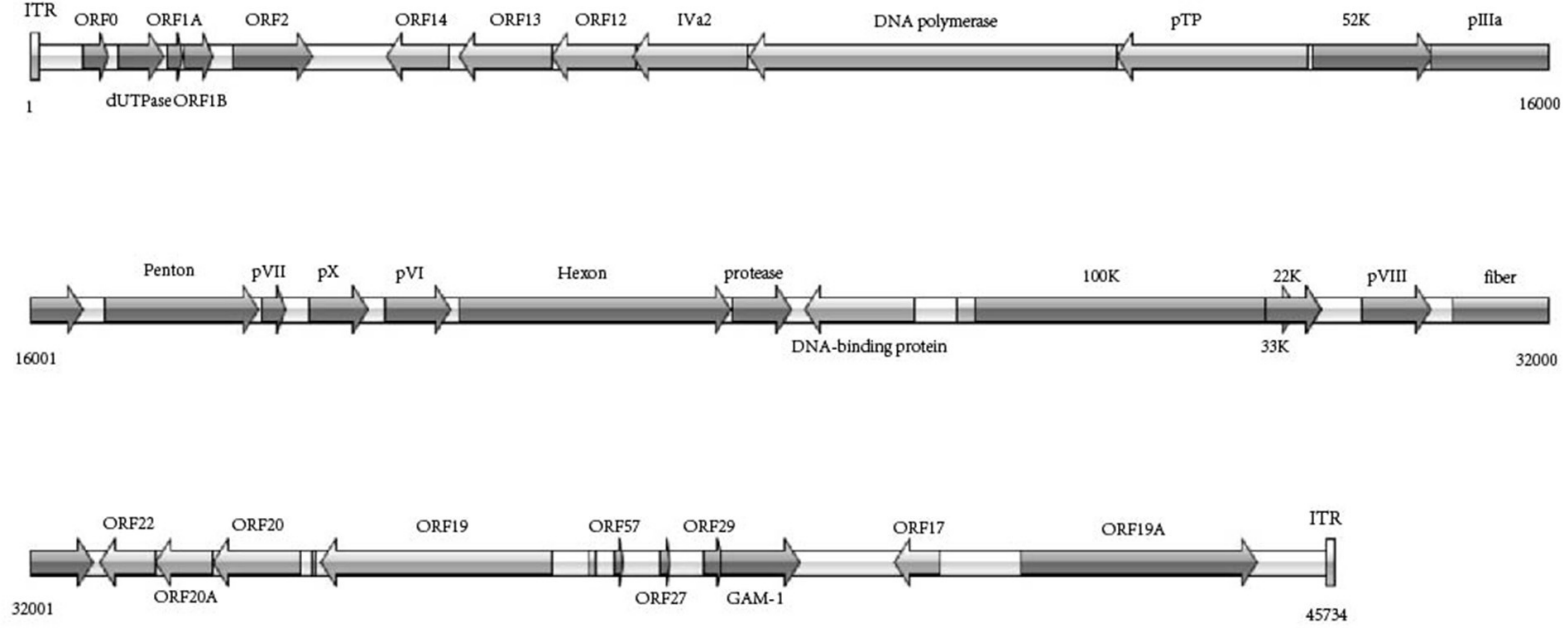
Schematic representation of genome size and organization of the FAdV-5 strain WHRS.

According to the phylogenetic analysis based on the genomic sequences, the WHRS strain was classified into a cluster that included FAdV-5 strain 340 (Figure 3A). The ORFs and terminal regions of the genomic sequence were similar to those of FAdV-5 340, including 35 potential ORFs (Figure 4). To determine the genetic diversity between the WHRS strain and other FAdV strains in the genus *Aviadenovirus*, a phylogenetic tree of the hexon proteins of FAdVs was constructed. The phylogenetic analysis of the hexon proteins showed that the WHRS strain was the most homologous to the FAdV-5 340 strain, sharing an amino acid identity of 97.5%. WHRS shared hexon amino acid sequence identity with other FAdV strains of 74.5~88.4%. The phylogenetic tree based on the hexon proteins clustered the FAdV strains into five clades, and the FAdV-5 strains WHRS and 340 were in an independent branch (FAdV-B) (Figure 3B). The deduced penton protein of WHRS showed 99.8% identity to that of 340. The similarity of penton protein between WHRS and other species of FAdVs ranged from 80.1 to 90.2% (Figure 3C).

### Pathogenicity of WHRS in ducks

**Figure 5:**
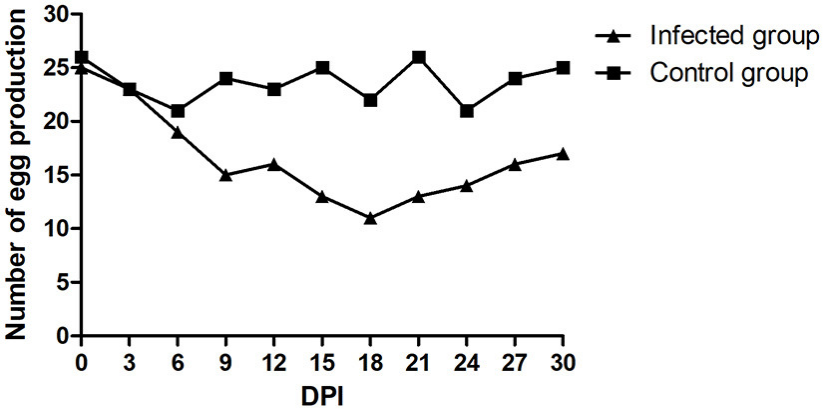
Daily egg production after the experimental infection.

There was no mortality in either the infected or control groups during the entire experimental procedure. No clinical signs were observed in the control group. The infected ducks showed loss of appetite, loose feathers and occasional thin stool. The egg production rate of the infected group declined during the entire monitoring period. The number of eggs produced was recorded daily. As shown in Figure 5, egg production in the infected group decreased to a minimum of 11/30 at 18 dpi but subsequently increased. The egg production rate of the control group was 70.0–86.7%. Viral nucleic acids were initially detected at 5 dpi and persisted in the feces of the infected group. FAdV-5 DNA was positively detected until 30 dpi. Simultaneously, the tests were continuously negative in the mock animals. The postmortem examination of infected ducks revealed mild liver swelling and necrotizing degenerated follicles that had not yet shed. Compared to those in the field samples, the lesions observed during necropsy of the infected ducks were mild. The livers and follicles were positive for FAdV-5 by PCR, and the FAdV strain could be re-isolated.

## Discussion

In recent years, egg drop syndrome cases in ducks have been reported in breeder duck flocks in China. In addition to stress and nutritional factors that may underlie the disease, many contagious agents, such as tembusu virus, influenza A virus, and EDSV, have been suggested to be the causative pathogens (24, 25). Duck hepatitis A virus can also cause egg drop syndrome symptoms (32). In our study, the egg laying rate gradually declined to 30–40% at 2 weeks and then increased to >80% but did not completely recover to the initial laying rate. The causative pathogen of duck egg drop syndrome has been suggested to be FAdV-5. Egg drop syndrome might be a serious threat to the duck breeder industry in China. Previous studies have reported that EDSV, which belongs to the genus *Atadenovirus*, can infect healthy hens and quail, leading to a drastic drop in egg production accompanied by the laying of abnormal eggs. EDSV was also isolated from ducks, geese, turkeys, and herring gulls (33–35). The FAdV-5 340 strain was originally identified in Ireland broiler samples early in the 1970s (21). FAdV-5 could cause various symptoms in broilers, such as hypoxia, acute cardiac decompensation, incomplete haemostasis, pulmonary oedema, and nephrosis. The necropsy results of the diseased broilers indicated fibrinous airsacculitis, pericarditis, enteritis, and arthritis.

The WHRS strain, considered to belong to the species FAdV-B, was more similar to the Fowl aviadenovirus B isolate 17/25702 and 14/24408, which were isolated from dead birds and broilers. Kaján et,al reported that most recent FAdV-B strains were isolated from dead birds during 2014–2018 and indicated the emergence of escape variants in FAdV-B. Till now, pathogenicity of FAdV-B to waterfowl is unclear The genomic characteristics of the WHRS strain were similar to those of the reference strain 340. However, it shared a low genomic sequence identity with other FAdVs and clustered into a distinct clade/subclade based on the phylogenetic analysis of genomic sequences and hexon and penton proteins. The fibers of FAdVs are considered to play an important role in the infectivity and pathogenicity of FAdVs (36). The FAdV-A and FAdV-C strains have two fiber genes, and most field isolates were highly pathogenic. The genome of FAdV-5 has a single fiber gene and exhibits lower pathogenicity.

Compared with in chickens, FAdVs show poor pathogenicity and lethality in ducks. A total of 4/16 dead ducks had a secondary bacterial infection, which might be a lethal factor in ducks. In the experimental test, the infected ducks showed only mild clinical symptoms, and no deaths occurred. The results suggest that duck flocks may be resistant to the disease in farms with good feeding conditions. We further investigated whether the WHRS strain was causative and observed a drop in egg production in breeder ducks. The severe drop in egg production of infected ducks was similar to that of naturally infected ducks. The egg production declined at 6 dpi in the two groups, which may have been due to stress. Egg production increased at 18 dpi, but viral DNA was detected. This result indicated that FAdV-5 infection was persistent in duck flocks. The low level of virus replication in ducks might be an important factor for the incomplete recovery of egg production.

In summary, the results of this study indicated that an outbreak of egg drop syndrome in breeder ducks was caused by the FAdV-5 WHRS strain. The disease showed low pathogenicity in the breeder ducks but severely reduced their egg production performance. Epidemiological surveys and pathogenicity tests of FAdV-5 are urgently needed.

## Data availability statement

Nucleotide sequence accession numbers have been deposited into the GenBank database under accession number: OM836676.

## Ethics statement

The animal study was reviewed and approved by Animal Ethics Committee of the Guangxi Veterinary Research Institute.

## Author contributions

ZX and JF designed and coordinated the study and helped to review the manuscript. HC performed the experiments, analyzed the data, and wrote the manuscript. ML analyzed the data and wrote the manuscript. SL, JK, and DL participated in the animal experiments. All authors contributed to the article and approved the submitted version.

## Funding

This study was supported by the Guangxi Ba Gui Scholars Program Foundation (2019A50), the National Natural Science Foundation of China (31702233), and the China Agriculture Research System (CARS-42-19).

## Conflict of interest

The authors declare that the research was conducted in the absence of any commercial or financial relationships that could be construed as a potential conflict of interest.

## Publisher's note

All claims expressed in this article are solely those of the authors and do not necessarily represent those of their affiliated organizations, or those of the publisher, the editors and the reviewers. Any product that may be evaluated in this article, or claim that may be made by its manufacturer, is not guaranteed or endorsed by the publisher.
