# Supplementary material for: Whole-genome sequence and pathogenicity of a fowl adenovirus 5 isolated from ducks with egg drop syndrome in China
Source: Front Vet Sci. 2022 Aug 12;9:961793. doi: 10.3389/fvets.2022.961793 (PMC9412081; doi:10.3389/fvets.2022.961793)
Supplement: Supplementary file 1 [file Table_1.DOCX]

**Table S1** Percent similarity of the nucleotide sequence of the whole genome of WHRS strain compared with Fav strains selected from GenBank

|  | FAV-B | FAV-A | TAdV-B | FAV-D | FAV-D | FAV-C | FAV-C | FAV-B | FAV-E | FAV-E | FAV-C | FAV-E | FAV-D | FAV-D | FAV-B | FAV-B |
| --- | --- | --- | --- | --- | --- | --- | --- | --- | --- | --- | --- | --- | --- | --- | --- | --- |
|  | WHRS | CELO | D90/2 | 685 | SR49 | KR5 | ON1 | 340 | CR119 | YR36 | SD1356 | TR59 | ON-NP2 | A-2A | 17/25702 | 14/24408 |
| WHRS | 100 | 46.0 | 41.4 | 63.0 | 61.5 | 38.0 | 38.0 | 85.7 | 61.7 | 62.0 | 61.5 | 61.6 | 62.9 | 63.2 | 99.95 | 99.95 |
